# Effect of green tea and mulberry leaf powders on the gut microbiota of chicken

**DOI:** 10.1186/s12917-019-1822-z

**Published:** 2019-03-06

**Authors:** Yuan Chen, Jiajia Ni, Hongwei Li

**Affiliations:** 1 School of Life Science, Huizhou University, Huizhou, 516007 China; 2 Department of Hepatobiliary Surgery II, Guangdong Provincial Research Center of Artificial Organ and Tissue Engineering, Zhujiang Hospital of Southern Medical University, Guangzhou, 510280 China; 3 State Key Laboratory of Organ Failure Research, Southern Medical University, Guangzhou, 510515 China

**Keywords:** Mulberry leaf powder, Green tea powder, Chicken, Gut microbiota

## Abstract

**Background:**

The gut microbiota is closely correlated with host health and is strongly influenced by food composition. Chinese herbs are usually used as natural feed additives in livestock production. Therefore, the present study assessed the influence of diet supplementation with green tea and mulberry leaf powders on the chicken gut microbiota. The gut microbiota compositions were determined using 16S rDNA sequencing.

**Results:**

Enhanced relative abundances of *Bacteroides, Prevotella*, and *Megamonas* were found in the chicken gut when mulberry leaf powder was added to diet. Conversely, a higher abundance of potentially pathogenic *Gallibacterium* was found in the chicken gut when the diet was supplemented with green tea powder. These results indicated that green tea powder and mulberry leaf powder can greatly affect the gut microbiota of chickens by changing their compositions.

**Conclusions:**

It is imperative to examine and evaluate the effects of Chinese herbs on animal health before they are introduced as feed additives in animal production.

## Background

The gut microbiota comprises the resident microorganisms in the digestive tract of the host. The gut microbiota is closely linked with host health and disease status [1–3]. In recent years, a large body of research has demonstrated that diet influences the composition of animal gut microbiota**.** Dogs fed on a natural diet have more diverse and abundant microbial compositions in the gut microbiota than dogs fed with commercial feed [4]. Raw meat-based diet influences fecal microbiome in healthy dogs [5]. Green tea powder in combination with a single strain of *Lactobacillus plantarum* was able to promote the growth of *Lactobacillus* in the intestine of C57BL/6J mice [6]. Essential oil supplementation exerts a positive effect on intestinal microbiota in Ross broilers [7].

As a safe alternative to antibiotics, many Chinese herbs are used as natural feed additives in livestock production [8, 9]. Among these natural feed additives, green tea and mulberry leaf are often used as feed additives in poultry [10–15]. Green tea is known to possess health-promoting properties [16–18]. Some studies have shown that green tea extracts selectively inhibit the growth of pathogenic bacteria, while showing no effect on the growth of beneficial bacteria [19–21]. Green tea powder, which is increasingly being used as a supplementary ingredient in foods, can affect gut microbiota in mice [6]. However, the impact of green tea powder as feed additive on gut microbiota has not been reported in poultry. Mulberry leaves and their extracts have been used in folk medicine due to their therapeutic properties, particularly for their anti-inflammatory, anti-diabetic, and antioxidant properties [22–24]. However, the effect of mulberry leaf on chicken gut microbiota has not been evaluated previously.

The use of next generation sequencing of 16S rRNA genes has greatly enhanced our understanding of the bacterial community present in the gastrointestinal (GI) tract of various animal species [25, 26]. In the present study, we performed 16S rDNA sequencing to investigate the effects of green tea powder and mulberry leaf powder on the gut microbiota compositions of chicken. Our results demonstrate that green tea powder and mulberry leaf powder can greatly affect the gut microbiota of chickens by changing its composition.

## Results

### Microbial diversity in the chicken gut

**Fig. 1:**
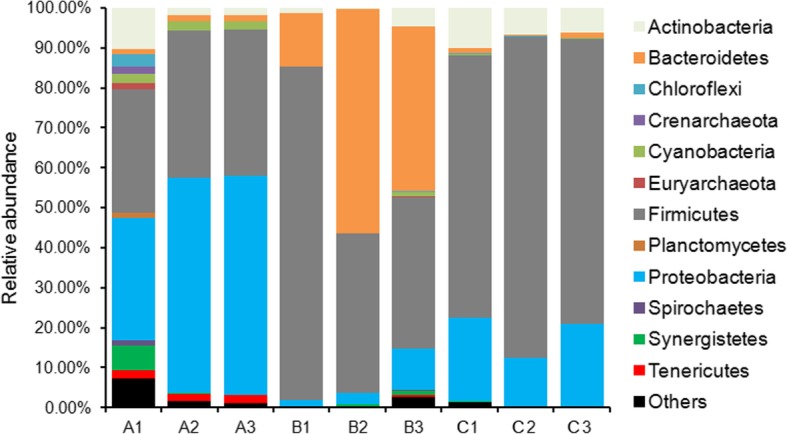
Dominant phyla in gut microbiota of chicken. Across all samples, total sequences were assigned to 41 phyla. The percentage bar diagram shows the composition of the dominant phyla in the chicken gut microbiota in different groups. Groups A, B, C represent three different treatments as follows: Group A was fed basal diet + 2% green tea powder; group B was fed basal diet + 4% mulberry leaf powder; group C was fed only basal diet as control. Each treatment was performed in three replicates (marked 1, 2, and 3)

To compare samples with different sequencing depths, each sample was rarefied to 8708 sequences. At a threshold of 97% sequence identity, 36,243 unique OTUs were identified in all samples. Across all samples, total sequences were assigned to 41 phyla (3 archaeal phyla and 38 bacterial phyla). *Firmicutes* (60.32 ± 21.96%), *Proteobacteria* (18.96 ± 17.99%), *Bacteroidetes* (11.55 ± 17.84%), *Actinobacteria* (4.50 ± 3.30%), *Synergistetes* (0.84 ± 1.61%), *Cyanobacteria* (0.72 ± 0.85%), *Tenericutes* (0.63 ± 0.93%), *Euryarchaeota* (0.41 ± 0.84%), *Chloroflexi* (0.31 ± 0.87%), *Acidobacteria* (0.28 ± 0.86%), *Spirochaetes* (0.20 ± 0.42%), *Crenarchaeota* (0.16 ± 0.49%), and *Planctomycetes* (0.13 ± 0.36%) were the dominant phyla across all samples. The composition of each sample at the phylum level is depicted in Fig. 1. Significant changes were observed among groups A, B, and C for alpha diversity of *Bacteroidetes* and *Proteobacteria*. Regarding alpha diversity of *Bacteroidetes*, there was a significant increase in group B compared to that in group C (One-way ANOVA, *P <* 0.019), whereas in case of *Proteobacteria*, a significant decrease was observed in group C compared to that in group A (One-way ANOVA, *P* < 0.001). In addition, *Tenericutes* was only found in group A. Cyanobacteria was clearly present in all replicates of group A, but it was only slightly represented in B3. These results indicate that green tea powder and mulberry leaf powder as feed additives in chicken diet greatly alter the alpha diversity of the chicken gut microbiota.

### Differences in gut microbial compositions among different groups

**Fig. 2:**
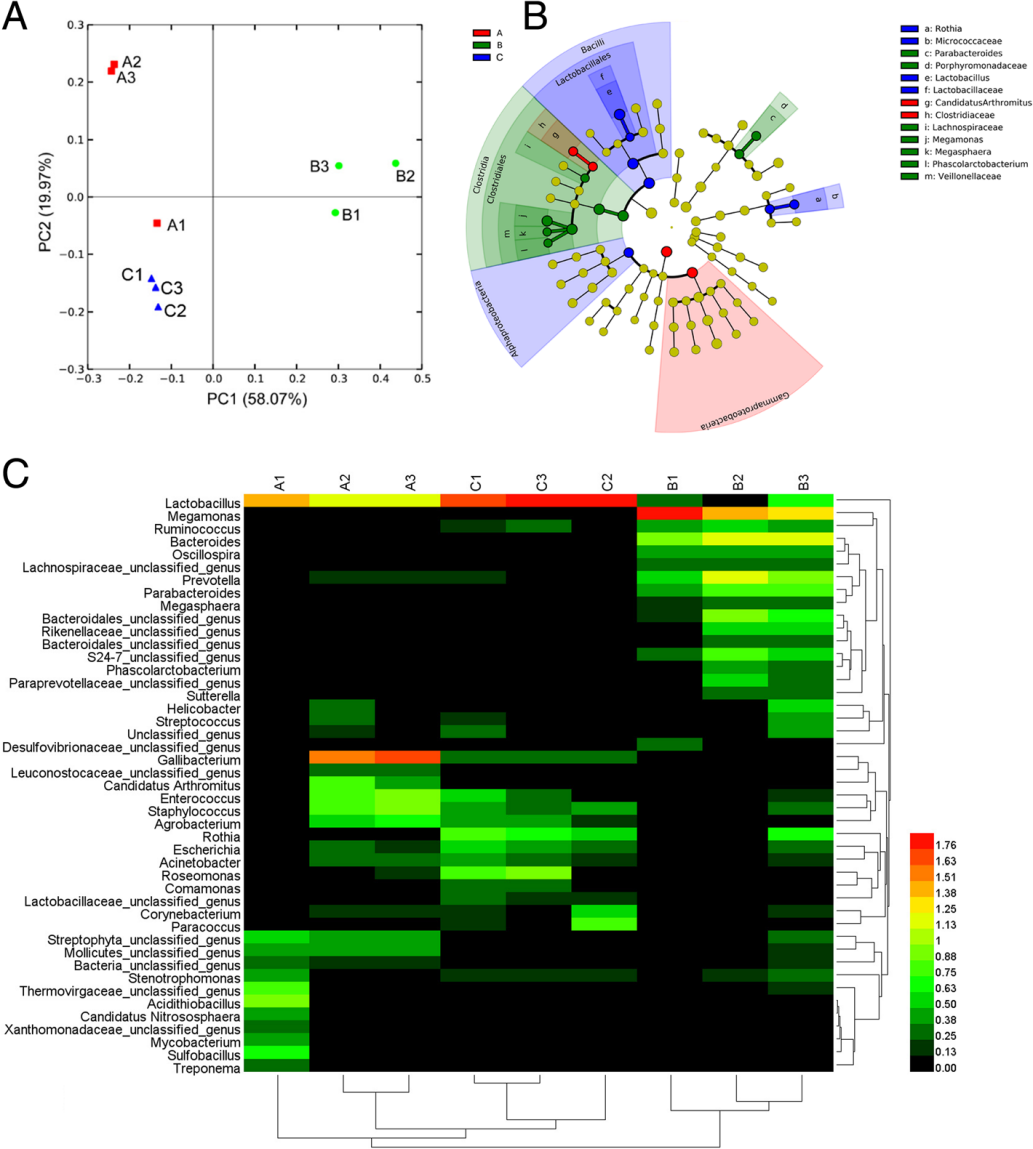
Gut microbiota differentiation of chicken with different feed additives. **a** PCoA analysis based on weighted UniFrac distance. Each point represents a sample. The first principal component is plotted on the X-axis, and the second principal component is plotted on the Y-axis. The colors indicate different groups. The percentage on each axis indicates the contribution to the discrepancy among samples. PCoA, principal coordinate analysis; Group A was fed basal diet + 2% green tea powder; group B was fed basal diet + 4% mulberry leaf powder; group C was fed only basal diet as control. **b** Phylogenetic profiles of specific bacterial taxa and predominant bacteria among the three different groups, as determined using the LEfSe analysis. Biomarker taxa are heighted by colored circles and shaded areas. Each circle’s diameter is relative to abundance of taxa in the community. **c** Group abundance heatmap showing normalized values of differentially abundant genera of the three groups. Group name is plotted on the X-axis, and the Y-axis represents the genus. Colors reflect relative abundance from low (green) to high (red)

Principal coordinate analysis (PCoA) was conducted based on weighted UniFrac distances to assess microbial distribution among the three groups. The weighted UniFrac plot showed that the gut microbial community of the A-B group was highly separated from that of the C group. In addition, a significant separation was observed between the feed additive groups (A-B group) and the non-feed additive group (C group) for PC1 and PC2 (58.07 and 19.97% of variance, respectively, *P* < 0.001) (Fig. 2a). The results indicated that the gut microbiota distribution in chicken was significantly influenced by the feed additives, similar to the distribution of alpha diversity.

Linear discriminant analysis (LDA) effect size (LEfSe) [27] was employed to identify specific phylotypes responding to feed additives in each group (Fig. 2b). LEfSe detected 13 bacterial taxonomic clades showing statistically significant differences among the three groups. At the family level, the relative abundances of *Porphyromonadaceae, Lachnospiraceae*, and *Veillonellaceae* were significantly increased in group B, while those of *Clostridiaceae*, and *Micrococcaceae* and *Lactobacillaceae* were significantly increased in groups A and C, respectively. At the genus level, *Parabacteroides, Megamonas*, *Megasphaera, and Phascolarctobacterium* were overrepresented in group B, while *Candidatus Arthromitus* was overrepresented in group A, and *Rothia and Lactobacillus* were overrepresented in group C. The results further showed that the feed additives, green tea powder and mulberry leaf powder, greatly affected the composition of the chicken gut microbiota.

HemI can be a useful toolkit for conveniently visualizing and manipulating heatmaps [28]. To provide a more visual view of the variation of the abundance of the dominant genera across samples, we used Hem1 software (see http://hemi.biocuckoo.org/download/HemI_Manual.pdf) to construct heatmaps (Fig. 2c). The results indicated that bacteria were significantly increased in the three groups. *Megamonas, Bacteroides*, and *Prevotella* had higher abundance in group B than in groups A and C, and *Lactobacillus* had higher abundance in groups A and C. This demonstrated that feed supplemented with mulberry leaf powder led to altered richness of bacteria compared to that without supplementation (C group).

## Discussion

### Effect of green tea powder on the gut microbiota of chicken

A recent study revealed that trillions of microorganisms live in the chicken gut, with the top four phyla being Firmicutes, Actinobacteria, Proteobacteria, and Bacteroidetes [29]. In consistent with this study, the top four phyla were also found in the chicken gut from different groups.

Microorganisms can benefit the host by aiding nutrient digestion and bioconversion of food chemicals, and abnormal changes in the gut microbiota could have undesirable effects on the health of the host [30]. Green tea is suggested to possess health-promoting properties [16–18]. Previous studies have demonstrated that green tea and its extracted products exert beneficial effects on chicken [10–12]. Recent studies have shown that green tea and its processed products alter gut microbiota composition in animals [6, 31]. Green tea powder, which includes both water- and non-water-soluble polyphenols as well as dietary fibers, is increasingly included as a supplementary ingredient in several food products. In the present study, green tea powder was used as a feed additive in chicken diet. The feed additive significantly affected bacterial diversity in the gut of chicken by promoting the prevalence of *Proteobacteria. Proteobacteria,* a major phylum of gram-negative bacteria, includes a wide variety of pathogens and many other notable genera [32]. Differences in the core microbiota at the family and genus levels were also observed between feed supplemented with mulberry leaf powder and the normal un-supplemented feed group. However, because of the limited sample size, further analyses are required to elucidate the intrinsic alterations in the gut microbiota when using green tea powder as a feed additive in chicken. Additional studies are necessary to determine the interaction between green tea powder as a feed additive and chicken health.

### Effect of mulberry leaf powder on the gut microbiota of chicken

Mulberry leaf is an important ingredient in some traditional Chinese medicinal formulations and is considered to have high nutritional value and antioxidant activity [24, 33]. It has been developed for use in functional food products. However, its effect on the gut microbiota of chicken is not known. In our study, mulberry leaf powder was used as feed additive in chicken diet to investigate its effect on chicken gut microbial diversity. We found that mulberry leaf powder could alter bacterial composition in the gut of chicken by improving the relative abundance of *Bacteroidetes*, *Bacteroides, Prevotella*, and *Megamonas*. *Bacteroidetes* is composed of three large classes of gram-negative bacteria and is widely distributed in the environment, including in the gut and on the skin of animals. Members of *Bacteroidetes* participate in providing the host with energy harvested from the diet through the fermentation of otherwise indigestible polysaccharides [34]. The three predominant *Bacteroidetes* genera of the human GI tract are *Bacteroides*, *Prevotella*, and *Porphyromonas*. A study indicated that *Prevotella* was more abundant in healthy children [35]. Further, an increase in *Bacteroides* may be attributed to reduced calorie load [36]. The two taxa, Bacteroides and Prevotella, are also considered “biomarkers” of diet and lifestyle in humans [37]. *Megamonas* is a genus of Firmicutes bacteria [38]. A previous study indicated that *Megamonas* acts as a hydrogen sink in the ceca of broilers by increasing the production of short chain fatty acids [39]. In the present study, the higher abundance of *Bacteroides, Prevotella*, and *Megamonas* in the chicken gut suggests that using mulberry leaf powder as feed additive in chicken could be beneficial for chicken health. However, because of the limited sample size, further research needs to be conducted to examine the interaction between mulberry leaf powder as a feed additive and chicken health.

## Conclusions

In conclusion, our study demonstrates that green tea powder and mulberry leaf powder can greatly change the composition of the chicken gut microbiota. However, the two herbal feed additives affected the gut microbiota in different ways, indicating that they may exert different and opposite effects on chicken health. Thus, it is imperative to examine and evaluate the effects of Chinese herbs on animal health before they are introduced as feed additives in animal production.

## Methods

### Animal population and study design

This study was performed by strictly following Animal management regulations of the People’s Republic of China. Healthy female Huiyang Bearded chickens were selected from the national Huiyang Bearded chicken breeding ground of Guangdong Jinzhong Agriculture and animal husbandry technology Co., Ltd. This poultry breed is a local broiler.

One hundred and twenty-day-old female chickens having similar body weights (1212.70 ± 24.25 g) were randomly divided into three treatment groups, with three replicates per treatment, and 10 birds per replicate. The trial was conducted in a screened shed environment with temperature variation from 22.5 to 30.5 °C at Guangdong Jinzhong Agriculture and animal husbandry technology Co., Ltd., from June 2017 to August 2017. Birds were housed in a commercial caging system (each cage being 40 × 40 × 30 cm in height, width, and depth, respectively). Chickens were randomly assigned to the cages, with three chickens in each unit. Water was supplied via two ‘on-demand’ nipples per cage. The three treatments comprised the following diets: group A was fed basal diet + 2% green tea powder (The dry Chinese green tea leaves were ground and sieved through a 0.5-mm sieve to obtain green tea powder), group B was fed basal diet + 4% mulberry leaf powder (Mulberry leaves were harvested in Bozhou city, China, lyophilized, and ground to powder using a vibrating sample mill), and group C was fed only basal diet and acted as the control. The basal diet consisted of 91.05% dry matter, metabolizable energy 12.96 MJ/kg, crude protein 16%, calcium 0.82%, and phosphorus 0.61% by dry weight. The whole experiment lasted 37 days. The chickens needed several days to adjust to the new breeding conditions before the formal experiment could be performed, so a preliminary experiment was necessary. However, the breeding conditions did not change in the chickens after the preliminary experiment. The duration of the preliminary experiment was 7 days and the duration of the formal experiment was 30 days. After the formal experiment, we randomly selected nine individuals for each treatment.

All chickens were euthanized by intravenous barbiturate overdose followed by cervical dislocation. Their gut contents were instantly collected from the ceca within 5 min of euthanasia, immediately placed in cryogenic vials, stored immediately at − 20 °C in a portable freezer, delivered to the laboratory and stored at − 80 °C until DNA extraction.

### DNA extraction, PCR, and 16S sequencing

The genomic DNA extraction kit for gut contents (TIANGEN Biotech, China) was used to extract total DNA of gut contents. The kit is based on silica membrane technology and provides special buffer system with InhibitEX Tablet for stool sample gDNA extraction (detailed procedure shown in TIANamp Stool DNA Kit Handbook, http://www.tiangen.com/asset/imsupload/up0044925001433136195.pdf). Nine DNA samples from each treatment was randomly divided into three pools to obtain three DNA samples per pool. DNA concentration and purity were determined using the Nanodrop 2000 Spectrophotometer. Amplification of the V4–V5 hypervariable region of the microbial 16S rRNA gene used the universal primers seen in [40]. 25 μL PCR amplification reaction mix included 1 × PCR buffer, 1.5 mM MgCl_2_, each primer at 1.0 μM, 0.25 U of Ex Taq (TaKaRa, China), and 10 ng genomic DNA. The PCR amplification procedure was as follow: denaturating at 94 °C for 3 min, followed by 30 cycles (every cycle consisted of denaturating at 94 °C for 40 s, annealing at 56 °C for 60 s, and elonging at 72 °C for 60 s), and a final extension at 72 °C for 10 min.

After PCR amplification, the two PCR products were mixed to run on 1.2% agarose gel. After the target band was excised, followed by purification using SanPrep DNA Gel Extraction Kit (Sangon Biotech, China). All amplicons were pooled together with an equal molar amount from each sample and sequenced using an Illumina MiSeq system at Guangdong Meilikang Bio-Science, Ltd., China.

### Bioinformatics and statistical analyses

The merged sequences were acquired by using the FLASH-software [41] to merging paired-end reads. In order to obtain clean data, the merged sequences were further analyszed by QIIME Pipeline-Version 1.9.0. The clean data were then filtered by chimera check by the Uchime algorithm [42]. After detection and removal of chimeras, the effective sequences were grouped into OTUs (Operational Taxonomic Units) at a user-defined level of sequence similarity (e.g., 97% to approximate species-level phylotypes). The representative sequences of each OTU were aligned to the core_set_aligned.fasta.imputed using align_seqs.py script in QIIME 1.9.0, and then the aligned sequences were filtered to remove gaps using filter_alignment.py script in the QIIME. The alpha diversity indices and weighted UniFrac distance metrics, which use phylogenetic information to calculate community similarity [43], were calculated through the QIIME pipeline. Taxonomy was assigned using the Ribosomal Database Project classifier [44]. Greengenes gg_13_8 (http://qiime.org/home_static/dataFiles.html) was used as a reference database. Statistical comparisons of microbial communities between treatments were determined using the linear discriminant analysis (LDA) effect size (LEfSe). LEfSe analysis was performed on the Galaxy website [27].

The original sequence data are available at the SRA by accession number PRJNA523073 (https://www.ncbi.nlm.nih.gov/sra/PRJNA523073).

## Abbreviations

GI: Gastrointestinal
LDA: Linear discriminant analysis
LEfSe: Linear discriminant analysis effect size
OTUs: Operational Taxonomic Units
PCoA: Principal coordinate analysis
